# Prevalence and characteristics of foreign body-induced upper gastrointestinal obstruction in dogs

**DOI:** 10.5455/javar.2025.l924

**Published:** 2025-06-03

**Authors:** Lalita Laiket, Wijit Sutthiprapa, Suchawalee Khattiya, Pimjai Temwichitr, Jatuporn Rattanasrisomporn, Naris Thengchaisri

**Affiliations:** 1 Surgical Unit, Kasetsart Veterinary Teaching Hospital, Bangkok, Thailand; 2 Department of Companion Animal Clinical Sciences, Faculty of Veterinary Medicine, Kasetsart University, Bangkok, Thailand

**Keywords:** Dogs, endoscopy, esophagus, foreign bodies, gastrointestinal obstruction, stomach

## Abstract

**Objectives:**

This study aimed to identify, categorize, and compare gastrointestinal foreign bodies in dogs, with a particular focus on those causing obstruction. The study also sought to distinguish between foreign body occurrences in the esophagus and the stomach, including the types of materials such as bones, plastic bags, fruit seeds, stones, and wires.

**Methods:**

A retrospective study was conducted on 261 dogs (151 males and 110 females) with foreign bodies lodged in the esophagus (*n =* 111) or stomach (*n =* 150). The sample consisted of 188 small dogs (<12 kg), 41 medium-sized dogs (12–24 kg), and 32 large dogs (>24 kg).

**Results:**

The study’s findings indicate a higher prevalence of gastric foreign bodies (57.47%) compared to esophageal foreign bodies (42.53%). Small breeds dominated esophageal cases (92.79%), with only a small percentage being medium breeds (7.21%). In contrast, gastric cases included a high number of small breeds (56.67%), as well as significant percentages of medium (22.00%) and large breeds (21.33%). Small-breed dogs were more likely to have foreign bodies in the esophagus, while larger dogs (medium and large breeds) were more likely to have them in the stomach (*p* < 0.001). Distinct material patterns emerged between the esophagus and stomach. Notably, the esophagus showed a higher incidence of bones (61.26%) and dried dog snacks (23.42%) compared to the stomach (2.67% and 0.00%, respectively). Conversely, the stomach exhibited a higher incidence of fabrics (20.00% *vs.* 1.80%), plant materials (18.67% *vs.* 4.50%), metallic objects (18.00% *vs.* 8.11%), rocks (12.67% *vs.* 0.9%), rubber materials (10.67% *vs.* 0.00%), plastic materials (6.67% *vs.* 0.00%), and hairballs (2.67% *vs.* 0.00%), respectively. A temporal analysis revealed that within the esophagus, 28.83% of cases underwent foreign body removal within 24 h, 56.76% within 2–7 days, and 14.41% after more than 7 days. For foreign bodies within the stomach, removal occurred in 23.33% of cases within 24 h, 30.00% within 2 to 7 days, 22.00% after more than 7 days, and 24.67% at an unknown timing. A total of 111 cases involved foreign bodies lodged in the esophagus, and 150 cases involved items stuck in the stomach. Endoscopic methods were primarily employed to remove foreign bodies, with surgical intervention required for 4 (3.60%) esophageal and 8 (5.30%) gastric cases, including noncrushable bones and resistant items such as rubber ducks. In dogs with complete follow-up, surgical removal of esophageal foreign bodies had a higher mortality rate (3/4, 75.00%) compared with endoscopic removal (3/56, 5.36%) (*p* < 0.002), while no mortality was observed in dogs with gastric foreign bodies undergoing surgical or endoscopic removal (*p* = 0.149).

**Conclusion:**

Esophageal foreign bodies were primarily composed of bones and dried dog snacks, while gastric foreign bodies more often contained fabrics, plant materials, and metallic objects. These composition differences highlight the need for site-specific management strategies.

## Introduction

Dogs’ tendency to ingest items indiscriminately puts them at risk of foreign body syndrome, a condition more prevalent in younger animals [1]. Dogs exhibiting pica or similar behaviors are particularly prone to ingesting foreign objects [2,3], resulting in complications such as gastric outflow obstruction, gastric perforation, or systemic illness due to the breakdown and absorption of foreign material. There is a significant incidence of gastrointestinal (GI) obstruction in dogs, primarily attributed to the ingestion of objects such as bones, plastic bags, fruit seeds, stones, and wires [4–6]. Occasionally, these objects are large or sharp, leading to impaction in the esophagus or stomach, with prolonged impaction [6] and sharp objects increasing the risk of adverse events, such as esophageal perforation, as seen with fishhook ingestion [4]. Reports on upper GI foreign bodies in dogs are limited, underscoring the need for further research on the types and prevalence of obstructive objects.

While GI endoscopy is the gold standard for removal, with over 95% success, further investigation is needed to refine management strategies that minimize complications and improve outcomes [7].

GI foreign bodies, often involving bony material such as chicken bones and mammalian ribs, pose a common canine emergency, presenting in both esophageal and gastric forms. Esophageal bone foreign bodies, constituting 30% to 80% of reported cases, demand urgent attention, with studies suggesting a higher risk of esophageal mucosal trauma and a poorer prognosis than other substances. Gastric bone foreign bodies receive less attention in research, with limited studies addressing their occurrence. Previous research has discussed injuries resulting from esophageal perforation and associated complications, including esophagitis, mucosal laceration, and esophageal stricture formation, a common complication [8,9]. Diagnostic methods include radiographs and esophagoscopy, and immediate removal of diagnosed esophageal foreign bodies is recommended [8]. The potential for gastric perforation is unlikely, and the higher digestibility of foreign bodies in the stomach, including bones, supports arguments for leaving them in place. Identifying patterns in the anatomical location of these foreign bodies is useful for gaining insights into the diversity and prevalence of GI obstruction in dogs and for developing targeted prevention strategies [10–12].

There has been a notable rise in incidents in Thailand [13] involving obstruction, where dogs ingest objects such as bones, plastic bags, fruit seeds, stones, and wires, some of which are sizeable or sharp and can result in impaction within the esophagus or stomach. The objective of this study was to identify and categorize the types of foreign bodies encountered in the upper GI systems of dogs, with a focus on differences between esophageal and gastric foreign bodies, their breed and sex distribution, the temporal aspects of foreign body removal, and the associated mortality rates for different removal methods.

## Materials and Methods

### Ethical approval

The current investigation underwent review and approval by the Ethics Committee of Kasetsart University (ID# ACKU67-VET-034), with written consent obtained from all dog owners participating in the study.

### Clinical examinations

The medical records of 261 dogs that had ingested foreign bodies were retrospectively analyzed at Kasetsart University’s Veterinary Endoscopy Unit over a 5-year period, from 2018 to 2022. Of these, 151 were male and 110 were female (Table 1). There were 188 small dogs (< 12 kg), 41 medium-sized dogs (12–24 kg), and 32 large dogs (> 24 kg). The survey documented instances of foreign bodies lodged in the esophagus for 111 dogs and in the stomach for 150 dogs, offering a comprehensive overview of the prevalence and distribution of foreign body ingestion in these animals.

The cases were categorized into three groups based on the timing of treatment at the clinic. Group A (67 cases) comprised extractions within 24 h of ingestion, with foreign bodies lodged in the esophagus in 32 cases (28.83%) and the stomach in 35 cases (30.97%). Group B (108 cases) encompassed extractions between 2 and 7 days after ingestion, with foreign bodies lodged in the esophagus in 63 cases (56.76%) and the stomach in 45 cases (39.82%). Group C (49 cases) involved extractions after more than 7 days, with foreign bodies lodged in the esophagus in 16 cases (14.41%) and the stomach in 33 cases (29.20%).

**Table 1.** Characteristics of dogs with a history of upper GI foreign bodies.

Characteristic	Esophagus	Stomach	*p*-value
*N* (%)	111 (42.53%)	150 (57.47)	
Sex, no. (%)			
Male	56 (50.45)	95 (63.33)	
Female	55 (49.55)	55 (36.67)	0.043
Age, years ± SD	4.87 ± 4.04	4.69 ± 4.53	0.740
Size, no. (%)			
Small (<12 kg)	103 (92.79)	85 (56.67)	
(12–24 kg)	8 (7.21)	33 (22.00)	
Large (>24 kg)	0 (0.00)	32 (21.33)	< 0.001

Abbreviation: SD, standard deviation.

### Radiographic findings

**Figure 1.**
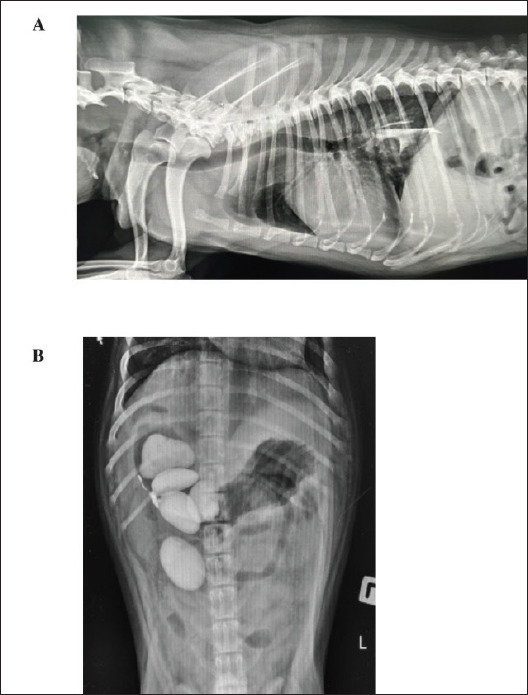
Radiographic images of foreign bodies in dogs with upper GI obstruction. A: LAT view; B: Dorsoventral view..

Thoracic and abdominal radiographs were obtained from multiple views, including lateral (LAT) and ventrodorsal (VD) positions (Fig. 1), to diagnose esophageal foreign bodies, which are typically caused by dietary indiscretion in dogs [14]. If a foreign body was suspected, blood and urine tests were conducted to assess the patient’s health status, evaluate potential complications from the obstruction, and determine the suitability of the dogs for anesthesia and endoscopic examinations.

### Endoscopic findings and removal procedures

**Figure 2.**
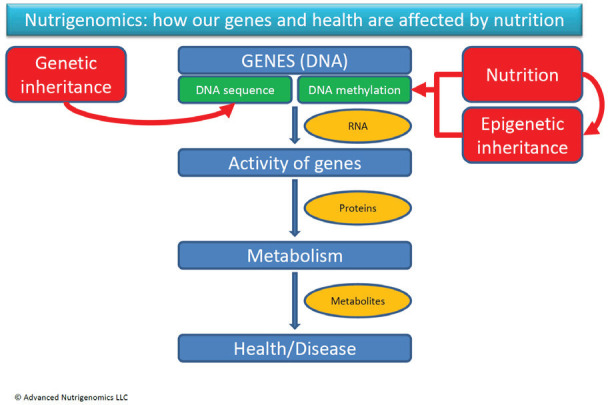
Foreign body retrieval instruments arranged from left to right: biopsy forceps, rat tooth forceps, stone basket, 3-prong grasping forceps, snare, and retrieval nets.

**Figure 3.**
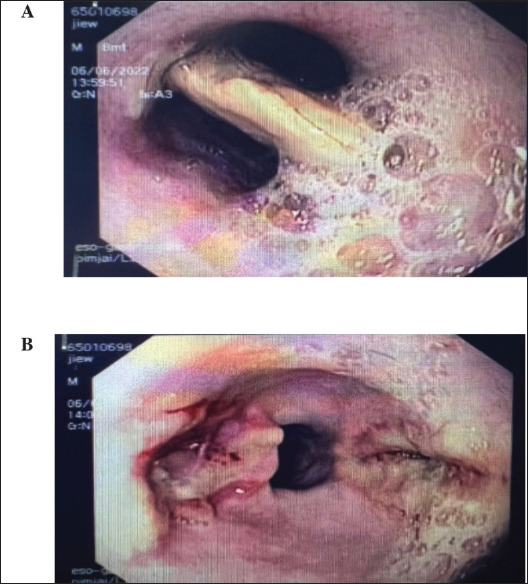
Endoscopic retrieval for esophageal foreign body obstruction. A: Chicken bone obstruction at the caudal part of the esophagus; B: Lesions of the esophageal mucosa after foreign body removal from the esophagus.

Administering sedation in dogs involves assessing their history, breed, sex, age, and laboratory test results [15]. After selecting an appropriate sedative, a flexible endoscope is smoothly inserted through the oral cavity, navigating the esophagus to the location of the suspected foreign object. Once in position, forceps are used to grasp and remove the foreign body. Endoscopic removal is preferred for GI foreign bodies, such as toys and coins, with surgery needed for more complex cases [16,17]. Following endoscopic identification, a range of retrieval instruments are used in sequence, including biopsy forceps, rat-tooth forceps, stone baskets, 3-prong grasping forceps, snares, and retrieval nets (Fig. 2). After extracting foreign bodies, any resulting lesions or tissue damage, such as abrasions or perforations, were documented in the medical record, often accompanied by photographs or diagrams to guide follow-up care (Fig. 3).

### Surgical interventions

In cases where endoscopic retrieval is unsuccessful or the object’s size prevents removal via endoscopy, surgical intervention becomes necessary to resolve the obstruction. Esophageal bone foreign bodies that cannot be managed through endoscopy are typically advanced into the stomach [18]. An incision along the midline of the abdominal wall is typically made. Upon gaining access to the abdomen, a comprehensive examination of the entire GI tract is conducted through direct visualization and palpation to precisely locate the foreign body. Following the extraction of the foreign body, dogs are transferred to the intensive care unit. Alternatively, due to limited facilities, some dogs may be referred to a nearby veterinary hospital at the owner’s request.

### Statistical analysis

The prevalence of upper GI foreign bodies in dogs was determined using commercially available statistical software packages (GraphPad Prism version 6.0, GraphPad Software, Inc., La Jolla, CA; STATA version 12, StataCorp, College Station, TX), and results are expressed as percentages. The relationship between various categorical parameters was assessed using Fisher’s exact test. Statistical significance was defined as *p* < 0.05.

## Results

In 261 dogs diagnosed with upper GI foreign bodies, 42.53% presented with esophageal foreign bodies, while 57.47% had gastric foreign bodies. The mean age of dogs presenting with esophageal foreign bodies (4.87 ± 4.04 years) did not differ significantly from that of dogs with gastric foreign bodies (4.69 ± 4.53 years, *p* = 0.740). A notable difference in breed distribution was observed between cases involving esophageal foreign bodies and those involving gastric foreign bodies (Table 1). Among esophageal occurrences, the majority (92.79%) were observed in small breeds, with a minority in medium-sized breeds (7.21%).

Conversely, cases associated with gastric foreign bodies showed a higher prevalence in small breeds (56.67%), with substantial proportions also found in medium (22.00%) and large breeds (21.33%). Small-breed dogs were associated with foreign bodies in the esophagus, while larger dogs (medium and large breeds) were associated with the foreign bodies in the stomach (*p* < 0.001). There was an approximately equal distribution of male (50.45%) and female (49.55%) dogs among cases with esophageal foreign bodies. However, a higher proportion of male dogs (63.33%) were affected by gastric foreign bodies compared to females (36.67%) (Table 1). Male dogs were associated with foreign bodies in the stomach (*p* = 0.043).

The diversity of foreign bodies discovered was extensive, encompassing bones (27.59%), metallic objects (13.79%), plant materials such as fruit seeds and wood pieces (12.64%), fabrics (12.26%), dried dog snacks (9.96%), rocks (7.66%), rubber materials (6.13%), plastic material (3.83%), hairballs (1.53%), and miscellaneous items (4.6 %) (Table 2). A higher incidence of foreign bodies was found in the esophagus than in the stomach, including bones (61.26% and 2.67%) and dried dog snacks (23.42% *vs.* 0.00%), respectively. On the other hand, a higher incidence of foreign bodies was found in the stomach compared to the esophagus, including fabrics (20.00% *vs.* 1.8%), plant materials (18.67% *vs.* 4.50%), metallic objects (18% *vs.* 8.11%), rocks (12.67% *vs.* 0.9%), rubber materials (10.67% *vs.* 0.00%), plastic materials (6.67% *vs.* 0.00%), and hairballs (2.67% *vs.* 0.00%), respectively (Table 2).

A temporal analysis of foreign body removal from the esophagus and the stomach was also conducted (Table 3). Among esophageal cases, 28.83% had the foreign body removed within 24 h, 56.76% within 2 to 7 days, and 14.41% after more than 7 days. For foreign bodies within the stomach, removal occurred for 23.33% of cases within 24 h, 30.00% within 2–7 days, 22.00% after more than 7 days, and 24.67% at an unknown timing (Table 3).

The extraction of foreign bodies initially involved an endoscopic approach, with surgical intervention considered in cases of unsuccessful retrieval (Table 4). In dogs with complete follow-up, three of the four dogs (75.00%) that underwent surgical removal of esophageal foreign bodies died (Table 4), representing a significantly higher mortality rate than that for dogs that underwent endoscopic removal of esophageal foreign bodies [3/56 (5.36%), *p* < 0.002] after endoscopic removal of esophageal foreign bodies. There was no significant association between the method of retrieval (endoscopic *vs.* surgical) and mortality rates in the stomach group (*p* = 0.149), as both techniques resulted in no deaths (Table 4).

**Table 2.** Characteristics of foreign bodies identified in the esophagus or stomach of dogs.

Foreign bodies	No. (%) of cases		
	Esophagus (*n =* 111)	Stomach (*n =* 150)	Total (*n =* 261)
Bones (chicken bone, fish bones)	68 (61.26)	4 (2.67)	72 (27.59)
Dried dog snacks (dried meat, raw high)	26 (23.42)	0 (0.00)	26 (9.96)
Fabrics (socks, fabric scraps)	2 (1.8)	30 (20.00)	32 (12.26)
Plant materials (fruit seeds, wood pieces)	5 (4.50)	28 (18.67)	33 (12.64)
Metallic objects (fishing hooks, coins)	9 (8.11)	27 (18.00)	36 (13.79)
Rocks (pebble, cement, brick)	1 (0.90)	19 (12.67)	20 (7.66)
Rubber materials	0 (0.00)	16 (10.67)	16 (6.13)
Plastic materials	0 (0.00)	10 (6.67)	10 (3.83)
Hairballs	0 (0.00)	4 (2.67)	4 (1.53)
Miscellaneous (mask, wet tissue, sponge)	0 (0.00)	12 (8.00)	12 (4.60)

**Table 3.** Timing of foreign body removal from the esophagus or stomach of dogs.

Timing of removal	No. (%) of cases		
	Esophagus (*n =* 111)	Stomach (*n =* 150)	Total (*n =* 261)
Within 24 h	32 (28.83)	35 (23.33)	67 (25.67)
Within 2–7 days	63 (56.76)	45 (30.00)	108 (41.38)
>7 days	16 (14.41)	33 (22.00)	49 (18.77)
Unknown	0 (0.00)	37 (24.67)	37 (14.18)

## Discussion

The results of this study offer new perspectives on the epidemiology and clinical management of GI foreign bodies in dogs, showcasing a wide range of ingested materials, including bones, plant matter, metals, and plastics. This study underscores the importance of vigilance regarding objects that dogs may ingest, spanning from household items to outdoor debris. The study’s findings indicate a higher prevalence of gastric foreign bodies (57.47%) compared to esophageal ones (42.53%), with small breeds being particularly prone to esophageal obstructions. While the mean age of affected dogs did not significantly differ between those with esophageal or gastric foreign bodies, a gender disparity was observed, with more male dogs affected by gastric foreign bodies (63.33%) than females (36.67%). Notable variations in ingested objects were highlighted, with bones and dried snacks more frequently lodged in the esophagus and fabrics and plant materials prevalent in the stomach. Timely endoscopic intervention was crucial for managing these cases, with surgical intervention only considered in specific situations.

**Table 4.** Techniques for retrieving upper GI foreign bodies from the esophagus or stomach of dogs with complete follow-up.

Locations	No. (%) of cases		*p*-value
	Endoscopic retrieval	Surgical retrieval	
Esophagus (*n =* 111)	107 (96.40)	4 (3.60)	
Loss to follow-up	51 (45.95)	0 (0.00)	
Death	3/56 (5.36)	3/4 (75.00)	0.002
Stomach (*n =* 150)	142 (94.70)	8 (5.30)	
Loss to follow-up	35 (23.33)	0 (0.00)	
Death	0/107 (0.00)	0/8 (0.00)	0.149

Both LAT and VD radiographic positions are essential for detecting and localizing foreign bodies in the upper GI tract [5]. The LAT view helps visualize objects in the neck and thoracic esophagus [14] and offers a clear side view of the stomach, while the VD view provides a broader abdominal view, helping identify objects in the midline or pelvic area and assess gas patterns. Bones and dried dog snacks were notably more prevalent in the esophagus than in the stomach, suggesting potential challenges in the passage of these items through the esophagus. However, these items can typically be digested by the stomach and do not pose significant risks once they reach this area. Conversely, fabrics, plant materials, metallic objects, rocks, rubber materials, plastic materials, and hairballs had a varied distribution, with a higher prevalence in the stomach, suggesting differences in the ease of passage or retention between these anatomical regions. Additionally, the materials, including fabrics, metallic objects, and plastics, are not digestible in the stomach’s acidic environment, highlighting the risks posed by such objects to canine GI health. These findings call for increased awareness about the potential dangers of ingesting certain materials, particularly non-digestible and sharp objects.

**Figure 4.**
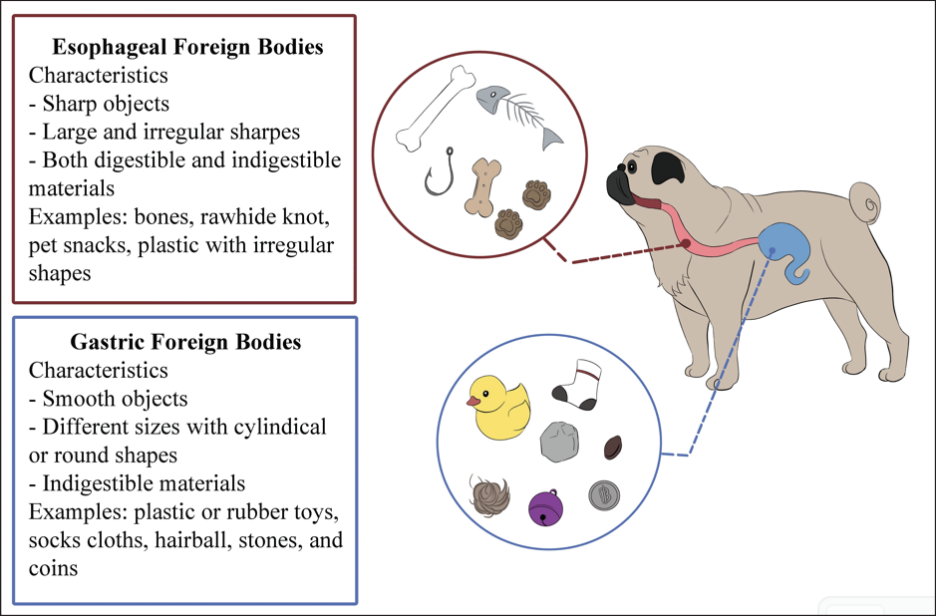
Common characteristics of esophageal and gastric foreign bodies identified in dogs.

Esophageal foreign bodies in dogs vary widely in size and shape, ranging from large or irregularly shaped items such as fishing hooks and bones to non-digestible materials such as synthetic leather bones and hard plastic toys (Fig. 4). Flexible endoscopy is a highly effective, minimally invasive method for foreign body removal in dogs, particularly in young animals, with a high success rate and low complication rate [19]. However, surgical management remains highly successful, especially when endoscopy fails or when perforation is present [20]. In this study, surgical intervention in the esophagus was necessary for 4 out of 111 cases, involving tightly lodged bones that were non-crushable and posed a risk of esophageal rupture. For gastric foreign bodies, surgical removal was required in 8 out of 150 cases where the objects, including rubber ducks and soccer balls, resisted crushing. One object could only be partially crushed due to size or density considerations [19,20].

Non-digestible foreign bodies carry significant risks, including perforation, obstruction, or injury to the esophageal lining, as well as the added risk of aspiration and airway blockage [21]. Conversely, gastric foreign bodies encompass a diverse array of characteristics, including organic substances such as fruit pits and bones, as well as inorganic entities such as hard plastic and cloth. Ranging in size from small entities such as thread to larger structures such as rubber toys and sticks, these foreign bodies, typically localized within the stomach, increase the risk of obstruction or injury (Fig. 4). The pressure exerted by these foreign bodies on the esophagus or stomach wall, coupled with stretching and bunching, can compromise tissue perfusion, necessitating prompt treatment to prevent shock in cases where endotoxins are released into the bloodstream [22].

Analysis of intervention timing in this study revealed varying timelines for foreign body removal. A higher proportion of foreign bodies were removed within the first week of the presentation, emphasizing the importance of swift intervention to minimize complications associated with GI foreign bodies. It should be noted that endoscopy offers an advantage over radiography and ultrasound by enabling tissue analysis and providing a curative option for cases of GI foreign bodies [21]. The present study identified a higher mortality rate associated with surgical removal of esophageal foreign bodies than endoscopic removal, underscoring the challenges and risks associated with invasive interventions in this anatomical location. Endoscopic methods for foreign body retrieval were minimally invasive and effective; nonetheless, surgical intervention is necessary in some cases (in this study, esophageal: 3.60%; stomach: 5.30%), particularly in cases involving non-crushable or resistant foreign bodies [23,24]. Notably, 75% of the dogs that underwent surgery for esophageal foreign body removal died, which is significantly higher than the 5.36% mortality rate for those treated with endoscopic removal. In contrast, none of the dogs that underwent surgical or endoscopic removal of gastric foreign bodies died. Therefore, it is important to carefully consider the type and location of foreign bodies when deciding on the appropriate management approach, particularly for esophageal foreign bodies.

In this study, a small percentage of dogs (3.83%) were observed ingesting plastics, which were retrieved via endoscopy or surgery. A postmortem study in Porto, Portugal, found microplastics in tissues from 35 animals (25 dogs and 24 cats), with most particles (1–10 µm) identified as polyethylene terephthalate (PET) [25]. Although the broader health impacts of plastic exposure remain poorly understood, recent research on PET microplastics indicates that serum-derived extracellular vesicles (EVs) can transport plastic particles. This process may alter miRNA content in EVs, potentially affecting genes associated with cardiovascular diseases, metabolic disorders, and cancer [26]. Chronic exposure to PET has been postulated to increase the risk of long-term health effects, including physical harm, chemical exposure, inflammation, behavioral changes in animals, and ecological disruptions [27], which emphasizes the need for interdisciplinary research to address these risks to both animal and human health.

The current study has several limitations. First, it was conducted at a single veterinary center in the Bangkok metropolitan area, which may limit the generalizability of the findings. This setting may not fully represent veterinary practices in different geographic areas. Second, the retrospective nature of the study may result in incomplete and inconsistent data due to reliance on historical records, potentially introducing biases. Third, this study did not examine intestinal foreign bodies, which may require different management. Additionally, prolonged foreign body entrapment and perforation might also occur, especially in rural areas, and these were associated with a poorer prognosis [28]. Alternatively, veterinarians can employ emetic drugs to remove certain gastric foreign bodies in dogs [29]. Despite these limitations, the study provides critical information on foreign bodies in the canine upper GI tract, which could help veterinary practitioners gain insights for developing preventive, diagnostic, and therapeutic strategies for occurring complications [30]. Future studies should involve multiple centers, extended follow-up, and exploration of behavioral factors such as pica, hyperactivity, and anxiety, which contribute to foreign body ingestion [1,2].

## Conclusion

The study of upper GI foreign bodies in dogs revealed that esophageal foreign bodies were most common in small breeds and mainly consisted of bones and dried dog snacks, while gastric foreign bodies were more varied, including fabrics, plant materials, and metallic objects. Endoscopic removal was successful in most esophageal cases, but surgery was required for unsuccessful retrieval, with a significantly higher mortality rate for surgical removal compared to endoscopy. Timely intervention is crucial to mitigate risks, preserve digestive tract integrity, and reduce complications, such as perforation and obstruction. The study did not examine intestinal foreign bodies, meaning that there is a need for broader research. Future studies should also explore behavioral factors and breed predispositions to improve owner awareness and develop preventive strategies to reduce foreign body ingestion in dogs.

## List of abbreviations

h, hours

kg, kilograms

μm, micrometer

EVs, extracellular vesicles

GI, gastrointestinal

LAT, lateral

miRNA, micro ribonucleic acid

PET, polyethylene terephthalate

VD, ventrodorsal.
